# The Study of the Expression of *CGB1* and *CGB2* in Human Cancer Tissues

**DOI:** 10.3390/genes11091082

**Published:** 2020-09-17

**Authors:** Piotr Białas, Aleksandra Śliwa, Anna Szczerba, Anna Jankowska

**Affiliations:** Department of Cell Biology, Poznan University of Medical Sciences, Rokietnicka 5D, 60-806 Poznań, Poland; glodek@ump.edu.pl (A.Ś.); abosacka@ump.edu.pl (A.S.); ajanko@ump.edu.pl (A.J.)

**Keywords:** human chorionic gonadotropin, TCGA, cancer, CGB1, CGB2

## Abstract

Human chorionic gonadotropin (hCG) is a well-known hormone produced by the trophoblast during pregnancy as well as by both trophoblastic and non-trophoblastic tumors. hCG is built from two subunits: α (hCGα) and β (hCGβ). The hormone-specific β subunit is encoded by six allelic genes: *CGB3*, *CGB5*, *CGB6*, *CGB7*, *CGB8*, and *CGB9*, mapped to the 19q13.32 locus. This gene cluster also encompasses the *CGB1* and *CGB2* genes, which were originally considered to be pseudogenes, but as documented by several studies are transcriptionally active. Even though the protein products of these genes have not yet been identified, based on The Cancer Genome Atlas (TCGA) database analysis we showed that the mutual presence of *CGB1* and *CGB2* transcripts is a characteristic feature of cancers of different origin, including bladder urothelial carcinoma, cervical squamous cell carcinoma, esophageal carcinoma, head and neck squamous cell carcinoma, ovarian serous cystadenocarcinoma, lung squamous cell carcinoma, pancreatic adenocarcinoma, rectum adenocacinoma, testis germ cell tumors, thymoma, uterine corpus endometrial carcinoma and uterine carcinosarcoma.

## 1. Introduction

Human chorionic gonadotropin (hCG) is a heterodimeric hormone, which comprises two non-covalently linked subunits: alpha (α) and beta (β). The α subunit is common to all gonadotropic hormones, such as follicle stimulating hormone (FSH), thyrotropin (TSH) and luteinizing hormone (LH), while the β subunit is hormone specific and determines its biological properties [1,2,3]. Most gonadotropins are synthesized by the anterior pituitary gland, however hCG synthesis takes place in the placenta as a result of syncytiotrophoblast cells activity [4].

Human chorionic gonadotropin participates in and regulates many physiological processes related to the normal course of pregnancy, which includes: maintenance of progesterone production by the corpus luteum, chorionic villi development, embryo implantation, or angiogenesis [5]. In addition, hCG determines the mother’s immunotolerance to the antigens presented by the developing fetus [6,7].

Human chorionic gonadotropin, especially its β subunit (hCGβ) is also secreted in gestational trophoblastic disease, and by a large group of non-trophoblastic tumors. hCGβ expression has been observed in tumors of different origin such as: breast, cervix, prostate, lung, colon, kidney, bladder, pancreas, anus, vulva, ovary, brain, endometrium and mouth [8,9].

The mechanisms regulating hCG and hCGβ expression, as well as their role in cancer development and growth, are not fully understood.

The formation of functional human chorionic gonadotropin is associated with the activation of genes encoding both subunits of the hormone (α and β). The α subunit is encoded by a single *CGA* gene, located on the long arm of the sixth chromosome at the 6q14-q21 locus. The β subunit is encoded by eight allelic genes: *CGB1*, *CGB2*, *CGB3*, *CGB5*, *CGB6*, *CGB7*, *CGB8*, and *CGB9*, located in the luteinizing hormone beta subunit/chorionic gonadotropin beta subunit (LHB/CGB) cluster on the long arm of the nineteenth chromosome at the 19q13.32 locus [10]. The cluster also holds the *CGB4* gene encoding the β subunit of luteotropic hormone [11,12].

Interestingly, sequencing of the nineteenth chromosome revealed that the *CGB7* and *CGB3* genes are allelic forms of the *CGB6* and *CGB9* genes, respectively [13,14].

The protein products of individual *CGB* genes show amino acid differences at position 117. This led to the division of the genes into two groups. The first group, which includes the *CGB7* and *CGB9* genes, was categorized as type I genes and gives rise to protein products with alanine in position 117. Type II genes, which include *CGB3*, *CGB5*, *CGB6*, and *CGB8*, encode a protein with aspartic acid in position 117 [10,12,15].

It is hypothesized that in the course of evolution, the *CGB3*, *CGB5*, *CGB6*, *CGB7*, *CGB8*, and *CGB9* genes arose as a result of multiple duplications of the gene encoding the β subunit of luteinizing hormone (*CGB4*) [16]. In contrast, the *CGB1* and *CGB2* genes evolved as a consequence of the insertion of a DNA fragment into the duplicated genes. In the case of the *CGB1* gene, this fragment included 736 base pairs, while for *CGB2*, it was 724 base pairs. The insertion led to the deletion of a 52-base long segment of the proximal promoter, as well as the entire 5′ untranslated region (5′UTR) region of the *CGB* gene. The consequence of this mutation was the creation of a new promoter sequence for *CGB1* and *CGB2*, a new 5′UTR region with an alternative start codon, and a new first exon. This led to a shift in the open reading frame for exons 2 and 3 [14,17]. The development of systemic and molecular biology methods made it possible to show that the nucleotide sequences of these genes in humans and chimpanzee differ by only 0.5% of their total composition. This high degree of conservatism of *CGB1* and *CGB2* confirms their common evolutionary path of origin [18].

Until recently, *CGB1* and *CGB2* were considered to be pseudogenes. Their biological activity was, however, demonstrated at the level of transcription. The genes’ transcripts have been detected in healthy tissues, such as placenta, testes and the pituitary gland [14,19,20,21,22]. *CGB1* and *CGB2* expression has also been confirmed in breast and bladder cancer cell lines, as well as in ovarian cancer tissue [9,23,24,25].

Although the contribution of *CGB1* and *CGB2* in the total expression of all *CGB* genes does not exceed 1/1000, both *CGB1* and *CGB2* genes were shown to have a considerable expression peak during the first trimester in normal pregnancy, but not in placentas of the second and third trimester, or in recurrent miscarriages. This particular expression pattern of the genes suggests their putative role in the implantation stage of the fetus development. Thus, it may be speculated that their expression drives placental cytotrophoblast cell invasion and growth during implantation. In the same manner, *CGB1* and *CGB2* may promote the pregnancy-like invasion of cancer cells into specific microenvironments, and later tumor growth and metastasis [26].

In order to verify the transcriptional activity of *CGB1* and *CGB2* genes in cancer, we analyzed data collected in TCGA.

## 2. Materials and Methods

Information related to thirty-three different types of tumors deposited in The Cancer Genome Atlas (TCGA) database was evaluated in terms of *CGB1* and *CGB2* gene expression. Twelve tumors, characterized by the presence of both *CGB1* and *CGB2* gene transcripts, were selected for further research and the expression level of the studied genes in cancer and corresponding non-malignant tissues was analyzed using UALCAN web-portal [27].

TCGA level 3 RNASeq V2 data equivalent to the expression of *CGB1* and *CGB2* in non-malignant and primary tumor samples for each gene was presented as a box and whisker plot. All values of gene expression were presented as transcripts per million (TPM)—a normalization method for RNA-seq which represents the relative abundance of a gene or transcript in a sample.

Student’s *t*-test was used to calculate the level of statistical significance (*p*-value). In the case of a lack of information regarding the expression of the genes or when the number of available data was extremely small, no statistical test was performed.

The data were presented using figures showing the interquartile range (IQR), including the median, minimum, maximum values, 25th percentile (Lower Q) and 75th (Upper Q) percentile (if available).

## 3. Results

To evaluate the expression level of *CGB1* and *CGB2* genes in cancers of different origin, the molecular characterization of thirty-three different tumors deposited in the TCGA database was analyzed. First, the screening of TCGA transcriptome data related to *CGB1* and *CGB2* gene expression in different cancers was performed.

**Figure 1:**
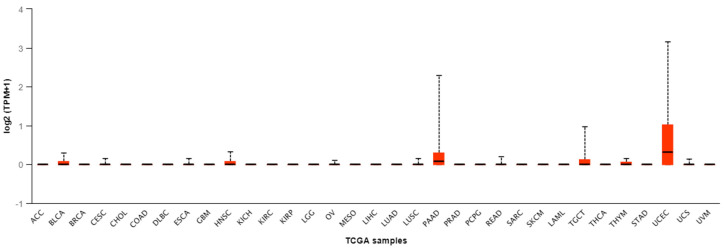
Expression level of *CGB1* in cancer, according to The Cancer Genome Atlas (TCGA) data; TPM—transcripts per million; ACC—Adrenocortical carcinoma; BLCA—Bladder urothelial carcinoma; BRCA—Breast invasive carcinoma; CESC—Cervical squamous cell carcinoma; CHOL—Cholangiocarcinoma; COAD—Colon adenocarcinoma; DLBC—Lymphiod neoplasm diffuse large B-cell lymphoma; ESCA—Esophageal carcinoma; GBM—Glioblastoma multiforme; HNSC—Head and Neck squamous cell carcinoma; KICH—Kidney chromophobe; KIRC—Kidney renal clear cell carcinoma; KIRP—Kidney renal papillary cell carcinoma; LGG—Brain lower grade glioma; OV—Ovarian serous cystadenocarcinoma; MESO—Mesothelioma; LIHC—Liver hepatocellular carcinoma; LUAD—Lung adenocarcinoma; LUSC—Lung squamous cell carcinoma; PAAD—Pancreatic adenocarcinoma; PRAD—Prostate adenocarcinoma; PCPG—Pheochromocytoma and Paraganglioma; READ—Rectum adenocacinoma; SARC—Sarcoma; SKCM—Skin cutaneous melanoma; LAML—Acute Myeloid Leukemia; TGCT—Testis germ cell tumors; THCA—Thyroid carcinoma; THYM—Thymoma; STAD—Stomach adenocarcinomna; UCEC—Uterine corpus endometrial carcinoma; USC—Uterine carcinosarcoma; UVM—Uveal Melanoma.

The *CGB1* gene transcript was found in 12 types of cancer only, namely: BLCA (Bladder urothelial carcinoma), CESC (Cervical squamous cell carcinoma), ESCA (Esophageal carcinoma), HNSC (Head and Neck squamous cell carcinoma), OV (Ovarian serous cystadenocarcinoma), LUSC (Lung squamous cell carcinoma), PAAD (Pancreatic adenocarcinoma), READ (Rectum adenocacinoma), TGCT (Testis germ cell tumors), THYM (Thymoma), UCEC (Uterine corpus endometrial carcinoma) and USC (Uterine carcinosarcoma); (Figure 1). The highest median values of *CGB1* expression were noted for two cancers: PAAD and UCEC (median: 0.078 and 0.317, respectively) (Table 1).

**Figure 2:**
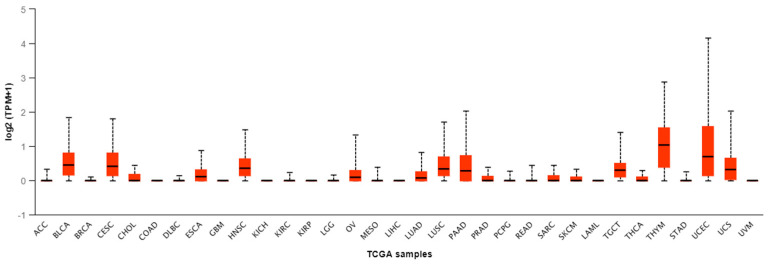
Expression of *CGB2* in cancer, according to TCGA data.

The *CGB2* gene transcripts were found in 26 types of cancer: ACC (Adrenocortical carcinoma), BLCA (Bladder urothelial carcinoma), BRCA (Breast invasive carcinoma), CESC (Cervical squamous cell carcinoma), CHOL (Cholangiocarcinoma), DLBC (Lymphiod neoplasm diffuse large B-cell lymphoma), ESCA (Esophageal carcinoma), HNSC (Head and Neck squamous cell carcinoma), KIRC (Kidney renal clear cell carcinoma), LGG (Brain lower grade glioma); OV (Ovarian serous cystadenocarcinoma), MESO (Mesothelioma), LUAD (Lung adenocarcinoma), LUSC (Lung squamous cell carcinoma), PAAD (Pancreatic adenocarcinoma); PRAD (Prostate adenocarcinoma), PCPG (Pheochromocytoma and Paraganglioma), READ (Rectum adenocacinoma), SARC (Sarcoma), SKCM (Skin cutaneous melanoma); TGCT (Testis germ cell tumors), THCA (Thyroid carcinoma), THYM (Thymoma), STAD (Stomach adenocarcinomna), UCEC (Uterine corpus endometrial carcinoma) and USC (Uterine carcinosarcoma); (Figure 2).

In BLCA, CESC, ESCA, HNSC, OV, LUAD, LUSC, PAAD, TGCT, THYM, UCEC, and USC, the median values of *CGB2* transcriptional activity were greater than 0, (median: 0.447, 0.412, 0.112, 0.356, 0.087, 0.084, 0.336, 0.279, 0.305, 1.033, 0.703, 0.318, respectively); (Table 2).

Among the 33 different studied cancers, only 12 were characterized by concomitant expression of *CGB1* and *CGB2.* This group consisted of BLCA, CESC, ESCA, HNSC, OV, LUSC, PAAD, READ, TGCT, THYM, UCEC and USC (Table 3). The highest TPM values noted for both studied *CGB* genes (*CGB1:* 3.158 and *CGB2*: 4.154) characterized UCEC (Table 3). Detailed analysis of the studied gene expression in these tumors revealed differences between cancerous and corresponding non-malignant tissues (Table 4).

**Figure 3:**
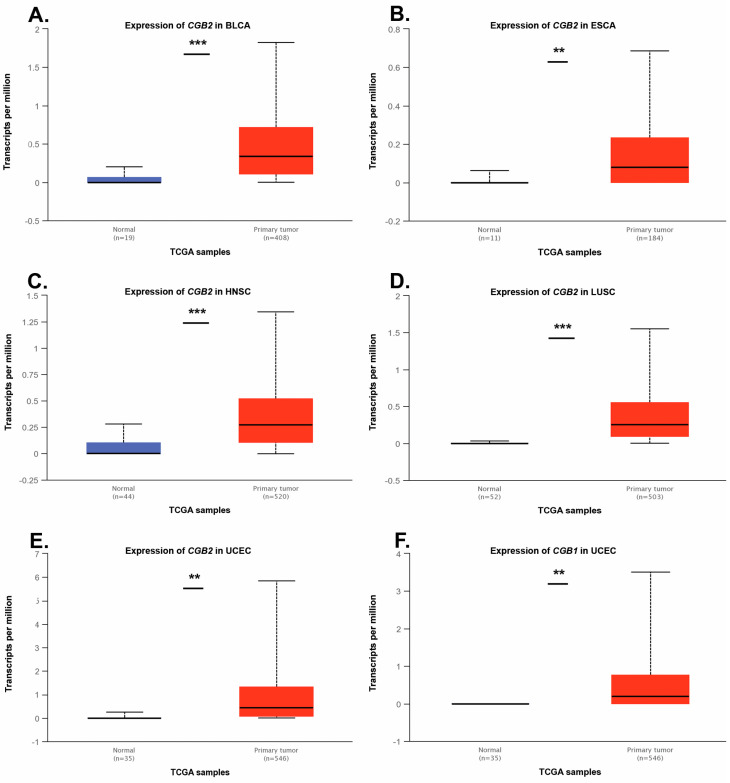
Expression levels of *CGB1* and *CGB2* in primary tumor samples and corresponding healthy tissues according to data deposited in the UALCAN database. (**A**) Expression of *CGB2* in BLCA, (**B**) expression of *CGB2* in ESCA, (**C**) expression of *CGB2* in HNSC, (**D**) expression of *CGB2* in LUSC, (**E**) expression of *CGB2* in UCEC, (**F**) expression of *CGB1* in UCEC. ** *p* ≤ 0.01; *** *p* ≤ 0.001.

Significant differences in *CGB2* expression in non-malignant tissues and primary tumor samples were noted for BLCA, ESCA, HNSC, LUSC and UCEC; *p*-value: 1 × 10^−6^, 0.006, <1 × 10^−12^, <1 × 10^−12^, 0.0017 respectively. The expression level of *CGB1* in primary tumor and healthy tissue differed for UCEC only (*p* = 0.0044) (Figure 3).

## 4. Discussion

The synthesis of the β subunit of human chorionic gonadotropin occurs as a result of the activity of the tissue-dependent genes mapped to chromosome 19 (19q13.3), called *CGB1*–*CGB9*. The expression level of individual *CGB* genes is not equal. The most transcriptionally active gene, with up to twenty times higher expression compared to other *CGB* genes in both placenta and many cancerous tissues, is *CGB5*. The genes with lower transcriptional activity are *CGB3*, *CGB6*, *CGB7*, *CGB8*, and *CGB9*, as well as *CGB1* and *CGB2* [18].

For a long time, the methods used for quantifying gene expression were insufficiently sensitive to detect the presence of *CGB1* and *CGB2* transcripts in cells and tissues. In fact, for this reason, the *CGB1* and *CGB2* genes were considered pseudogenes.

Although the expression levels of *CGB1* and *CGB2* are a thousand times lower compared to the remaining *CGBs*, it has been shown that these genes’ transcripts play an important role in chorionic villi invasion and embryo implantation [26]. It was suggested that their expression may drive both placental cytotrophoblast cell invasion, as well as the invasion of cancer cells.

Indeed, it was reported that in bladder cancer cell lines, it is *CGB2* expression, but not other *CGB* genes, which correlates with the amount of functional free hCGβ protein secreted by cancer cells and with cancer growth. It was also demonstrated that specific targeting of *CGB1* and *CGB2* with siRNA was much more effective in reducing cancer cell numbers than silencing other *CGB* genes [28]. Thus, it was suggested that *CGB1* and *CGB2* products give rise to tumor-driving molecules.

Since the transcriptional activity of *CGB1* and *CGB2* in cancer was mainly shown in cell lines, the aim of the present study was to demonstrate the expression of these genes in human cancer tissues of different origin. The data deposited in TCGA database was used in order to verify *CGB1* and *CGB2* transcriptional activity status in malignant and corresponding non-malignant tissue.

The results of the study showed that out of thirty-three available records related to the studied genes’ expression, *CGB1* transcriptional activity characterized twelve types of cancer only, namely: BLCA, CESC, ESCA, HNSC, OV, LUSC, PAAD, READ, TGCT, THYM, UCEC, and USC.

At the same time *CGB2* transcripts were detected in twenty six cancers, including: ACC, BLCA, BRCA, CESC, CHOL, DLBC, ESCA, HNSC, KIRC, LGG, OV, MESO, LUAD, LUSC, PAAD, PRAD, PCPG, READ, SARC, SKCM, TGCT, THCA, THYM, STAD, UCEC, and USC.

Interestingly, only twelve cancer types were characterized by the concomitant expression of *CGB1* and *CGB2.* This group included bladder urothelial carcinoma, cervical squamous cell carcinoma, esophageal carcinoma, head and neck squamous cell carcinoma, ovarian serous cystadenocarcinoma, lung squamous cell carcinoma, pancreatic adenocarcinoma, rectum adenocacinoma, testis germ cell tumors, thymoma, uterine corpus endometrial carcinoma and uterine carcinosarcoma.

Despite the fact that the studied genes’ expression levels in cancer tissues were low, with the median often equaling zero, the transcriptional activity of the *CGB1* and *CGB2* genes in cancerous tissues of different origin was confirmed. Significant differences between *CGB2* expression levels in healthy and cancerous tissues were shown. Such differences, which characterized bladder urothelial carcinoma, esophageal carcinoma, head and neck squamous cell carcinoma, lung squamous cell carcinoma, and uterine corpus endometrial carcinoma, may have diagnostic implications. In the case of the *CGB1* gene, such a difference was recognized for uterine corpus endometrial carcinoma only. The analysis of the effect of *CGB1* and *CGB2* gene expression on patient survival did not show any significant correlation.

As mentioned earlier, *CGB1* and *CGB2* gene products are believed to drive placental cytotrophoblast cell invasion and growth during implantation. In the same way, they may promote the pregnancy-like invasion of cancer cells into specific microenvironments, and later tumor growth and metastasis. In fact, the results of TCGA database analysis show that a crucial factor in carcinogenesis may be *CGB2* gene expression. The gene’s products were detected in 26 out of 33 studied samples, and statistically significant differences in *CGB2* expression between malignant and non-malignant tissues were noted.

However, taking into account the limited data available, it cannot be excluded that both *CGB1* and *CGB2* gene products drive tumor development.

Further studies are needed in order to verify the hypothesis that *CGB1* and *CGB2* genes are involved in tumor growth, invasion and metastasis.

## Figures and Tables

**Table 1:** Expression values of *CGB1* in cancer samples deposited in TCGA. Q—quartile.

Tumor Type	Number of Samples	Minimal Value	Lower Q	Median	Upper Q	Maximal Value
ACC	79	0	0	0	0	0
BLCA	408	0	0	0	0.071	0.297
BRCA	1097	0	0	0	0	0
CESC	305	0	0	0	0	0.159
CHOL	36	0	0	0	0	0
COAD	286	0	0	0	0	0
DLBC	48	0	0	0	0	0
ESCA	184	0	0	0	0	0.152
GBM	156	0	0	0	0	0
HNSC	520	0	0	0	0.075	0.321
KICH	67	0	0	0	0	0
KIRC	533	0	0	0	0	0
KIRP	290	0	0	0	0	0
LGG	513	0	0	0	0	0
OV	305	0	0	0	0	0.103
MESO	87	0	0	0	0	0
LIHC	371	0	0	0	0	0
LUAD	515	0	0	0	0	0
LUSC	503	0	0	0	0	0.152
PAAD	178	0	0	0.078	0.302	2.291
PRAD	497	0	0	0	0	0
PCPG	179	0	0	0	0	0
READ	166	0	0	0	0	0.198
SARC	260	0	0	0	0	0
SKCM	472	0	0	0	0	0
LAML	173	0	0	0	0	0
TGCT	150	0	0	0	0.124	0.997
THCA	505	0	0	0	0	0
THYM	120	0	0	0	0.061	0.149
STAD	415	0	0	0	0	0
UCEC	546	0	0	0.317	1.016	3.158
USC	57	0	0	0	0	0.134
UVM	80	0	0	0	0	0

**Table 2:** Expression values of *CGB2* in different tumor types according to TCGA.

Tumor Type	Number of Samples	Minimal Value	Lower Q	Median	Upper Q	Maximal Value
ACC	79	0	0	0	0	0.329
BLCA	408	0	0.157	0.447	0.8	1.848
BRCA	1097	0	0	0	0	0.105
CESC	305	0	0.133	0.412	0.801	1.81
CHOL	36	0	0	0	0.17	0.44
COAD	286	0	0	0	0	0
DLBC	48	0	0	0	0	0.144
ESCA	184	0	0	0.112	0.311	0.869
GBM	156	0	0	0	0	0
HNSC	520	0	0.15	0.356	0.631	1.475
KICH	67	0	0	0	0	0
KIRC	533	0	0	0	0	0.236
KIRP	290	0	0	0	0	0
LGG	513	0	0	0	0	0.158
OV	305	0	0	0.087	0.299	1.329
MESO	87	0	0	0	0	0.396
LIHC	371	0	0	0	0	0
LUAD	515	0	0	0.084	0.257	0.823
LUSC	503	0	0.14	0.336	0.69	1.71
PAAD	178	0	0	0.279	0.725	2.024
PRAD	497	0	0	0	0.132	0.38
PCPG	179	0	0	0	0	0.265
READ	166	0	0	0	0	0.434
SARC	260	0	0	0	0.145	0.442
SKCM	472	0	0	0	0.098	0.329
LAML	173	0	0	0	0	0
TGCT	150	0	0.106	0.305	0.502	1.414
THCA	505	0	0	0	0.105	0.288
THYM	120	0	0.384	1.033	1.537	2.871
STAD	415	0	0	0	0	0.257
UCEC	546	0	0.135	0.703	1.572	4.154
USC	57	0	0.033	0.318	0.644	2.019
UVM	80	0	0	0	0	0

**Table 3:** The comparison of *CGB1* and *CGB2* expression in non-malignant and cancerous tissues according to The Cancer Genome Atlas (TCGA) database data.

Tumor Type	Number of Samples	Gene	Minimal Value	Lower Q	Median	Upper Q	Maximal Value
BLCA	408	*CGB1*	0	0	0	0.071	0.297
		*CGB2*	0	0.157	0.447	0.8	1.848
CESC	305	*CGB1*	0	0	0	0	0.159
		*CGB2*	0	0.133	0.412	0.801	1.81
ESCA	184	*CGB1*	0	0	0	0	0.152
		*CGB2*	0	0	0.112	0.311	0.869
HNSC	520	*CGB1*	0	0	0	0.075	0.321
		*CGB2*	0	0.15	0.356	0.631	1.475
OV	305	*CGB1*	0	0	0	0	0.103
		*CGB2*	0	0	0.087	0.299	1.329
LUSC	503	*CGB1*	0	0	0	0	0.152
		*CGB2*	0	0.14	0.336	0.69	1.71
PAAD	178	*CGB1*	0	0	0.078	0.302	2.291
		*CGB2*	0	0	0.279	0.725	2.024
READ	166	*CGB1*	0	0	0	0	0.198
		*CGB2*	0	0	0	0	0.434
TGCT	150	*CGB1*	0	0	0	0.124	0.997
		*CGB2*	0	0.106	0.305	0.502	1.414
THYM	120	*CGB1*	0	0	0	0.061	0.149
		*CGB2*	0	0.384	1.033	1.537	2.871
UCEC	546	*CGB1*	0	0	0.317	1.016	3.158
		*CGB2*	0	0.135	0.703	1.572	4.154
USC	57	*CGB1*	0	0	0	0	0.134
		*CGB2*	0	0.033	0.318	0.644	2.019

**Table 4:** The difference in *CGB1* and *CGB2* expression in non-malignant and primary tumor tissues based on data deposited in TCGA database. *p* ≤ 0.05 was considered as statistically significant. n.a.—not applicable.

Tumor Type	Number of Tissue Samples		Gene	*p*
	Non-Malignant	Primary Tumor		
BLCA	19	408	*CGB1*	0.07
			*CGB2*	1 × 10^−6^
CESC	3	305	*CGB1*	n.a.
			*CGB2*	n.a.
ESCA	11	184	*CGB1*	n.a.
			*CGB2*	0.006
HNSC	44	520	*CGB1*	n.a.
			*CGB2*	<1 × 10^−12^
OV	no data	305	*CGB1*	n.a.
			*CGB2*	n.a.
LUSC	52	503	*CGB1*	n.a.
			*CGB2*	<1 × 10^−12^
PAAD	4	178	*CGB1*	n.a.
			*CGB2*	n.a.
READ	10	166	*CGB1*	n.a.
			*CGB2*	n.a.
TGCT	no data	150	*CGB1*	n.a.
			*CGB2*	n.a.
THYM	2	120	*CGB1*	n.a.
			*CGB2*	n.a.
UCEC	35	546	*CGB1*	0.0044
			*CGB2*	0.0017
USC	no data	57	*CGB1*	n.a.
			*CGB2*	n.a.

## Abbreviations

ACC	Adrenocortical carcinoma
BLCA	Bladder urothelial carcinoma
BRCA	Breast invasive carcinoma
CESC	Cervical squamous cell carcinoma
CHOL	Cholangiocarcinoma
COAD	Colon adenocarcinoma
DLBC	Lymphoid neoplasm diffuse large B-cell lymphoma
ESCA	Esophageal carcinoma
GBM	Glioblastoma multiforme
HNSC	Head and Neck squamous cell carcinoma
KICH	Kidney chromophobe
KIRC	Kidney renal clear cell carcinoma
KIRP	Kidney renal papillary cell carcinoma
LGG	Brain lower grade glioma
OV	Ovarian serous cystadenocarcinoma
MESO	Mesothelioma
LIHC	Liver hepatocellular carcinoma
LUAD	Lung adenocarcinoma
LUSC	Lung squamous cell carcinoma
PAAD	Pancreatic adenocarcinoma
PRAD	Prostate adenocarcinoma
PCPG	Pheochromocytoma and Paraganglioma
READ	Rectum adenocarcinoma
SARC	Sarcoma
SKCM	Skin cutaneous melanoma
LAML	Acute Myeloid Leukemia
TGCT	Testis germ cell tumors
THCA	Thyroid carcinoma
THYM	Thymoma
STAD	Stomach adenocarcinoma
UCEC	Uterine corpus endometrial carcinoma
USC	Uterine carcinosarcoma
UVM	Uveal Melanoma
Mdn	Median
